# Vascular endothelial growth factor polymorphisms are associated with osteosarcoma susceptibility

**DOI:** 10.18632/oncotarget.10278

**Published:** 2016-06-24

**Authors:** Yuan-Yuan Hu, Xin-Ya Du, Ai-Ling Zhan, Lan Zhou, Qian Jiang, Yu-Ming Niu, Ming Shen

**Affiliations:** ^1^ Department of Stomatology, Taihe Hospital, Hubei University of Medicine, Shiyan 442000, China; ^2^ Department of Stomatology, People's Hospital of New District Longhua Shenzhen, Shenzhen 518109, China; ^3^ Department of Anesthesiology, Central Hospital of Shanghai Songjiang District, Shanghai 201600, China; ^4^ Department of Neurology, Taihe Hospital, Hubei University of Medicine, Shiyan 442000, China; ^5^ Center for Evidence-Based Medicine and Clinical Research, Taihe Hospital, Hubei University of Medicine, Shiyan 442000, China; ^6^ Jiangsu Key Laboratory of Oral Diseases, Department of Dental Implant, Affiliated Hospital of Stomatology, Nanjing Medical University, Nanjing 210029, China

**Keywords:** vascular endothelial growth factor, osteosarcoma, polymorphism, meta-analysis

## Abstract

Polymorphisms in the vascular endothelial growth factor (*VEGF*) gene may contribute to osteosarcoma risk, but the results of previous studies have been inconsistent and inconclusive. We conducted a meta-analysis to assess this association more accurately. Relevant studies were collected systemically from three online English databases. Crude odds ratios (ORs) and 95% confidence intervals (CIs) were used to assess the strength of the associations of three *VEGF* gene polymorphisms (+936C/T, '634 G/C, +1612 G/A) with osteosarcoma risk. Seven case-control studies involving 1,350 cases and 1,706 controls were selected for the meta-analysis. The pooled OR indicated that the *VEGF* +936C/T polymorphism was associated with increased risk of osteosarcoma in a Chinese population (T vs. C: OR = 1.26, 95% CI = 1.12–1.42, *P* < 0.01; TT vs. CC: OR = 1.70, 95% CI = 1.29–2.24, *P* < 0.01; CT + TT vs. CC: OR = 1.23, 95% CI = 1.06–1.44, *P* < 0.01; TT vs. CC + CT: OR = 1.61, 95% CI = 1.23–2.10, *P* < 0.01). A significant association was also found between the −634 G/C polymorphism and osteosarcoma risk (C vs. G: OR = 0.81, 95% CI = 0.69-0.96, *P* = 0.01; CC vs. GG: OR = 0.66, 95% CI = 0.48–0.90, *P* < 0.01; GC + CC vs. GG: OR = 0.80, 95% CI = 0.67–0.96, *P* = 0.02; CC vs. GG + GC: OR = 0.72, 95% CI = 0.60–0.86, *P* < 0.01). In sum, our meta-analysis suggests *VEGF* polymorphisms are associated with osteosarcoma susceptibility in the Chinese population. However, further studies that include different ethnicities and larger populations are needed.

## INTRODUCTION

Osteosarcoma, one of the most common types of malignant primary bone tumors, is characterised by the direct formation of immature bone or osteoid tissue by tumor cells, and is most prevalent in children and young people [1, 2]. In the 1970s, adjuvant chemotherapy for osteosarcoma was introduced to supplement standard treatments such as surgery and radiotherapy, and resulted in a high five-year survival rate [3, 4]. However, the survival of patients with initial pulmonary metastases and recurrent disease remained low [5]. Other side effects, such as bone disability, physical dysfunction and drug toxicity of chemotherapy, seriously reduce patients' quality of life, bringing a heavy medical burden to their families and the society [6]. To date, the pathogenesis of osteosarcoma remains unclear.

Recent molecular studies have suggested that genome dysfunction contributes to tumorigenesis [7, 8]. Mutations and abnormal expression of certain genes, such as those encoding microRNA, interleukin family proteins and cytochrome P450 superfamily members, facilitate the development of tumors and other malignant diseases [9–12]. Angiogenesis is a critical process to promote tumor cell proliferation and metastasis through the formation of new capillaries [13, 14]. Vascular endothelial growth factor (VEGF) is an important stimulator of physiological and pathological angiogenesis that acts on vascular endothelial cells and promotes human blood vessel growth during tumor formation and growth, enabling invasion and metastasis [15–17]. Increased VEGF expression has been detected in tumor tissue, blood and urine samples from patients with esophageal, prostate, lung, and other cancers.[18–21].

*VEGF* is located at chromosome 6p21.3 and comprises a 14-kb coding region with eight exons and seven introns [22, 23]. Several single-nucleotide polymorphisms (SNPs) have been described for the VEGF gene [24, 25]. Some of these polymorphisms, including those in the promoter and 5′-untranslated region, have been shown to alter the expression and biological activity of VEGF [26]. In 2014, Wang et al reported the first case-control study showing that +936 C/T was associated with a significant increase in osteosarcoma risk [27]. To date, the three most common *VEGF* polymorphisms, namely +936C/T (rs3025039), +1612G/A (rs10434), and −634G/C (rs2010963), have been investigated for their association with osteosarcoma risk, but the results have been inconsistent. Therefore, we conducted a comprehensive meta-analysis of all published studies to determine the association between *VEGF* polymorphism and osteosarcoma susceptibility more accurately. Our meta-analysis was performed according to the Preferred Reporting Items for Systematic Reviews and Meta-Analyses (PRISMA) statement [28, 29]. No ethical issues were involved in this study, given that our data were based on published studies.

## RESULTS

### Study characteristics

**Figure 1:**
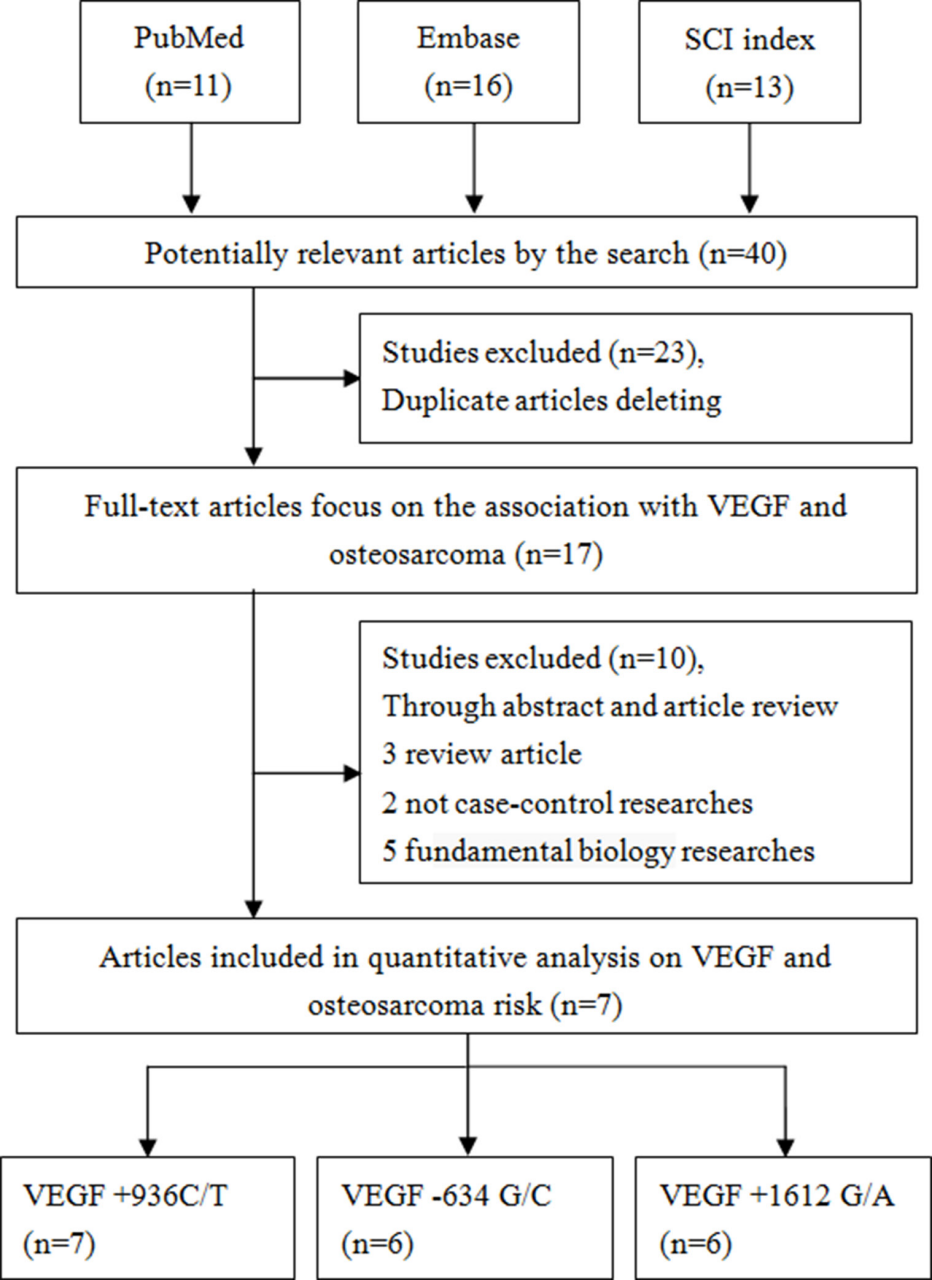
Flow diagram of the study selection process

Forty studies were identified through literature searching. In accordance with the selection criteria described in the Methods, 23 studies were excluded for duplication in the first step of title screening, and 17 studies were excluded for other reasons (two were review articles, five were not case-control studies, and eight were fundamental biology studies) during the systematic screening. Ultimately, seven articles were included in this meta-analysis (Figure 1, Table 1), all of which involved Chinese populations [27, 30–35]. For the +936C/T polymorphism, the seven studies included a total of 1,350 cases and 1,706 controls [27, 30–35]. For the +1612 G/A polymorphism, the six studies included a total of 1,166 cases and 1,524 controls [27, 30–32, 34, 35]. For the −634 G/C polymorphism, the six studies involved a total of 1,166 cases and 1,524 controls [27, 30–32, 34, 35]. All the included case-control studies used the polymerase chain reaction-restriction fragment length polymorphism (PCR-RFLP) method. In three studies of the +936C/T polymorphism, the genotype distribution of the controls deviated from Hardy-Weinberg equilibrium (HWE) [30, 33, 34].

**Table 1:** Characteristics of case-control studies on VEGF polymorphisms and osteosarcoma risk included in the meta-analysis

First author	Year	Control design	Genotype method	Case	Control	Genotype distribution						P for HWE^a^	MAF	
						Case			Control				Case	Control
+936C/T						CC	CT	TT	CC	CT	TT			
Wang	2014	Population-based	PCR-RFLP	330	342	185	116	29	207	123	12	0.22	0.26	0.21
Tie	2014	Hospital-based	PCR-RFLP	165	330	111	39	15	232	74	24	< 0.01	0.21	0.18
Zhao	2015	Hospital-based	PCR-RFLP	176	176	85	75	16	92	71	13	0.89	0.30	0.28
Zhang2	2015	Population-based	PCR-RFLP	180	360	66	92	22	148	175	37	0.16	0.38	0.35
Zhang1	2015	Hospital-based	PCR-RFLP	182	182	128	35	19	138	32	12	< 0.01	0.20	0.15
Liu	2015	Hospital-based	PCR-RFLP	186	186	125	46	16	134	42	10	0.01	0.21	0.17
Hu	2015	Hospital-based	PCR-RFLP	130	130	67	47	16	79	44	7	0.79	0.30	0.22
−634 G/C						GG	GC	CC	GG	GC	CC			
Wang	2014	Population-based	PCR-RFLP	330	342	115	165	50	118	166	58	0.98	0.40	0.41
Tie	2014	Hospital-based	PCR-RFLP	165	330	43	80	42	59	151	120	0.34	0.50	0.59
Zhao	2015	Hospital-based	PCR-RFLP	176	176	30	85	61	28	81	67	0.67	0.59	0.61
Zhang2	2015	Population-based	PCR-RFLP	180	360	42	90	48	53	170	138	0.96	0.52	0.62
Liu	2015	Hospital-based	PCR-RFLP	186	186	45	91	50	31	86	69	0.63	0.51	0.60
Hu	2015	Hospital-based	PCR-RFLP	130	129	42	68	20	46	65	18	0.51	0.42	0.39
+1612 G/A						**GG**	**GA**	**AA**	**GG**	**GA**	**AA**			
Wang	2014	Population-based	PCR-RFLP	330	342	95	157	78	97	172	73	0.84	0.47	0.46
Tie	2014	Hospital-based	PCR-RFLP	165	330	68	76	20	151	146	33	0.79	0.35	0.32
Zhao	2015	Hospital-based	PCR-RFLP	176	176	77	80	19	80	78	18	0.87	0.34	0.32
Zhang2	2015	Population-based	PCR-RFLP	180	360	77	80	23	163	155	42	0.58	0.35	0.33
Liu	2015	Hospital-based	PCR-RFLP	186	186	75	86	25	84	83	19	0.82	0.37	0.33
Hu	2015	Hospital-based	PCR-RFLP	130	130	41	61	28	46	60	24	0.57	0.45	0.42

a HWE in control.

MAF: Minor allele frequency.

### Quantitative analysis

### For the +936C/T polymorphism

**Figure 2:**
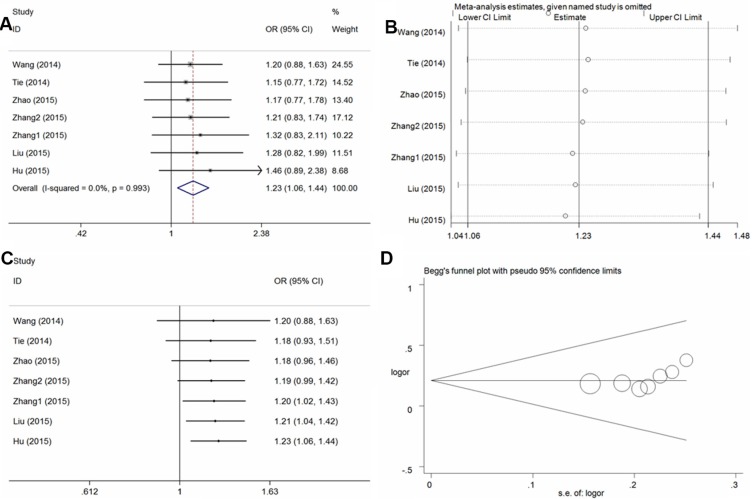
Statistical analysis of the association between the *VEGF* +936C/T polymorphism and osteosarcoma risk in the CT + TT vs. CC model (**A**) ORs and 95% CIs; (**B**) sensitivity analysis; (**C**) cumulative analysis; (**D**) publication bias.

In the seven studies examined, a significant increase in osteosarcoma risk was observed in four genetic models (T vs. C: Odds Ratio [OR] = 1.26, 95% Confidence Interval [CI] = 1.12–1.42, *P* < 0.01, *I*^2^ = 0%; TT vs. CC: OR = 1.70, 95% CI = 1.29–2.24, *P* < 0.01, *I*^2^ = 0%; CT+TT vs. CC: OR = 1.23, 95% CI = 1.06–1.44, *P* < 0.01, *I*^2^ = 0% [Figure 2A], TT vs. CC+CT: OR = 1.61, 95% CI = 1.23–2.10, *P* < 0.01, *I*^2^ = 0%) (Table 2). Similarly increased risk also detected in the subgroup analysis of the control from hospital population (T vs. C: OR = 1.28, 95% CI = 1.09–1.50, *P* < 0.01, *I*^2^ = 0%; TT vs. CC: OR = 1.62, 95% CI = 1.15–2.30, *P* < 0.01, *I*^2^ = 0%; CT+TT vs. CC: OR = 1.26, 95% CI = 1.03–1.53, *P* = 0.02, *I*^2^ = 0%, TT vs. CC+CT: OR = 1.55, 95% CI = 1.10–2.19, *P* = 0.01, *I*^2^ = 0%). Sensitivity analyses were performed by excluding each single study one by one, and the results of pooled ORs still presented stability (Figure 2B for the dominant model). Moreover, cumulative analysis with publication date demonstrated that the osteosarcoma risk was increasing gradually and presented positive with the report by Zhang et al. in 2015 (Figure 2C for the dominant model). Funnel plot symmetry was performed to estimate publication bias, and no evidence of asymmetry was obtained (Figure 2D for the dominant model). The results were further validated by Egger's test (T vs. C, *P* = 0.26; TT vs. CC, *P* = 0.28; CT+TT vs. CC, *P* = 0.11; TT vs. CC+CT, *P* = 0.24).

**Table 2:** Summary ORs and 95% CI of VEGF polymorphisms and osteosarcoma risk

	N*	T vs. C				CT vs. CC				TT vs. CC				CT+TT vs. CC				TT vs. CC+CT			
+936C/T		OR	95% CI	*P*	*I*^2^ (%)	OR	95% CI	*P*	*I*^2^ (%)	OR	95% CI	*P*	*I*^2^ (%)	OR	95% CI	*P*	*I*^2^ (%)	OR	95% CI	*P*	*I*^2^ (%)
Total	7	**1.26**	**1.12–1.42**	**< 0.01**	**0**	1.14	0.97–1.34	0.12	0	**1.70**	**1.29–2.24**	**< 0.01**	**0**	**1.23**	**1.06–1.44**	**< 0.01**	**0**	**1.61**	**1.23–2.10**	**< 0.01**	**0**
HWE-yes	4	**1.25**	**1.08–1.45**	**< 0.01**	**0**	1.13	0.93–1.38	0.22	0	**1.82**	**1.27–2.60**	**< 0.01**	**14.6**	**1.23**	**1.02–1.49**	**0.03**	**0**	**1.68**	**1.19–2.37**	**< 0.01**	**28.1**
HWE-no	3	**1.27**	**1.04–1.57**	**0.02**	**0**	1.15	0.87–1.52	0.34	0	**1.54**	**1.00–2.37**	**0.05**	**0**	1.24	0.97–1.59	0.09	0	1.49	0.97–2.28	0.07	0
Design																					
HB	5	**1.28**	**1.09–1.50**	**< 0.01**	**0**	1.16	0.94–1.44	0.17	0	**1.62**	**1.15–2.30**	**< 0.01**	**0**	**1.26**	**1.03–1.53**	**0.02**	**0**	**1.55**	**1.10–2.19**	**0.01**	**0**
PB	2	**1.23**	**1.03–1.47**	**0.03**	**0**	1.10	0.86–1.41	0.43	0	1.85	0.93–3.71	0.08	55.6	1.20	0.95–1.52	0.12	0	1.75	0.81–3.75	0.15	66.2
−634 G/C		C vs. G				GC vs. GG				CC vs. GG				GC + CC vs. GG				CC vs. GG + GC			
Total	**6**	**0.81**	**0.69–0.96**	**0.01**	**54.2**	0.88	0.82–1.06	0.18	0	**0.66**	**0.48–0.90**	**< 0.01**	**46.2**	**0.80**	**0.67–0.96**	**0.02**	**33.9**	**0.72**	**0.60–0.86**	**< 0.01**	**5.9**
Design																					
HB	4	0.82	0.66–1.01	0.07	51.9	0.86	0.66–1.13	0.28	0	0.67	0.44–1.01	0.06	44.1	0.78	0.61–1.01	0.06	31.5	**0.72**	**0.57–0.91**	**< 0.01**	**12.7**
PB	2	0.80	0.56–1.15	0.23	78.4	0.86	0.57–1.29	0.46	50.3	0.63	0.32–1.25	0.19	74.5	0.76	0.44–1.31	0.33	74.2	0.71	0.48–1.05	0.09	46.5
+1612 G/A		A vs. G				GA vs. GG				AA vs. GG				GA + AA vs. GG				AA vs. GG + GA			
Total	6	1.10	0.98–1.23	0.10	0	1.07	0.91–1.27	0.40	0	1.21	0.95–1.54	0.12	0	1.11	0.94–1.30	0.21	0	1.17	0.95–1.46	0.15	0
Design																					
HB	4	1.14	0.98–1.33	0.10	0	1.13	0.90–1.41	0.28	0	1.31	0.93–1.83	0.12	0	1.17	0.94–1.44	0.16	0	1.22	0.90–1.67	0.21	0
PB	2	1.06	0.89–1.25	0.52	0	1.00	0.77–1.30	0.98	0	1.11	0.79–1.57	0.54	0	1.04	0.81–1.32	0.78	0	1.13	0.84–1.53	0.42	0

* Numbers of comparisons.

*I2* for Heterogeneity test.

### For the −634 G/C polymorphism

**Figure 3:**
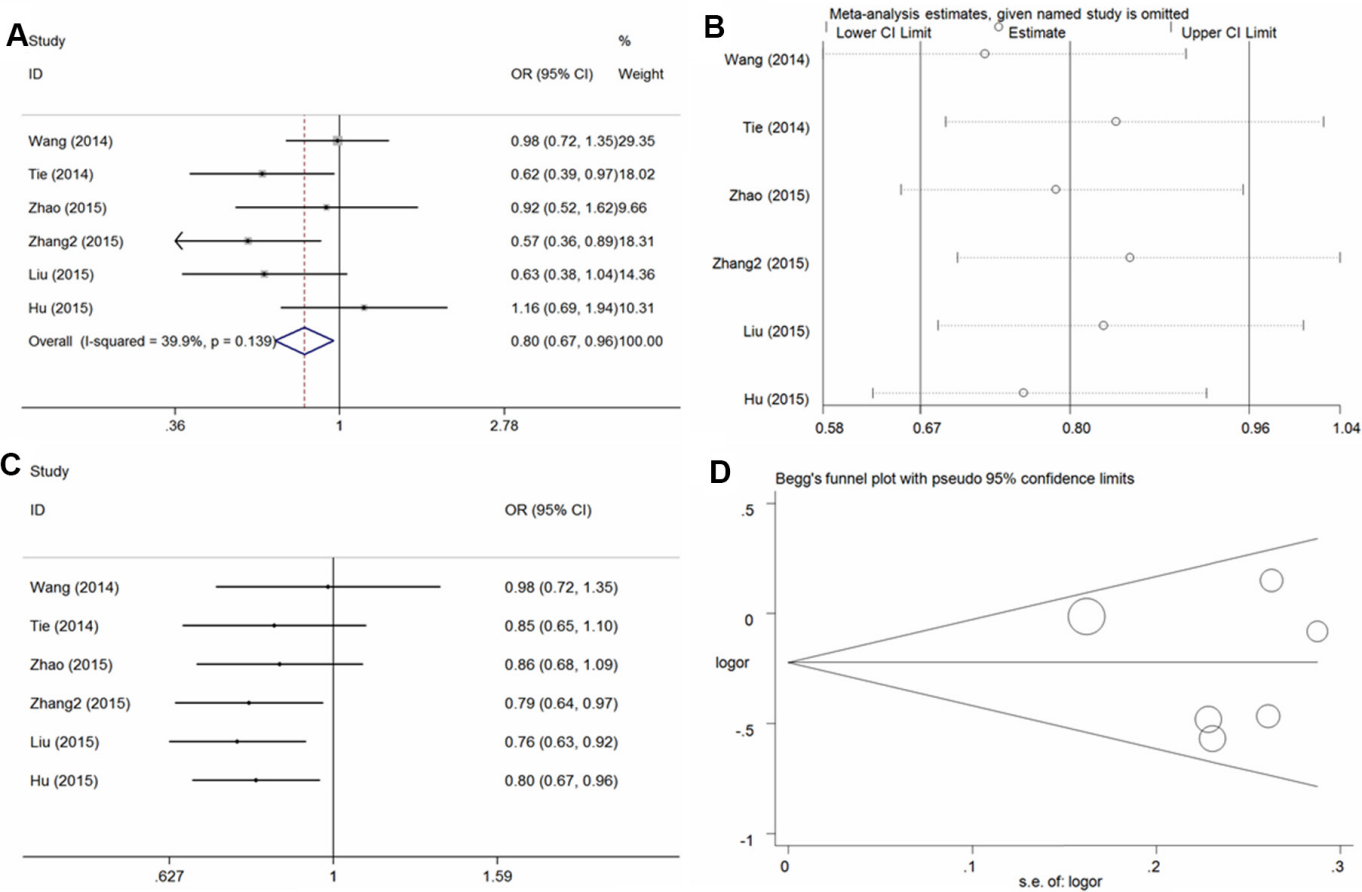
Statistical analysis of the association between the *VEGF* − 634G/C polymorphism and osteosarcoma risk in the GC + CC vs. GG model (**A**) ORs and 95% CIs; (**B**) sensitivity analysis; (**C**) cumulative analysis; (**D**) publication bias.

In the six studies examined, overall, significant protective effects of the −634 G/C polymorphism against osteosarcoma risk were found in the four genetic models (C vs. G: OR = 0.81, 95% CI = 0.69–0.96, *P* = 0.01, *I*^2^ = 54.2%; CC vs. GG: OR = 0.66, 95% CI = 0.48–0.90, *P* < 0.01, *I*^2^ = 46.2%; GC+CC vs. GG: OR = 0.80, 95% CI = 0.67–0.96, *P* = 0.02, *I*^2^ = 33.9% (Figure 3A for the dominant model); CC vs. GG+GC: OR = 0.72, 95%CI = 0.60-0.86, *P* < 0.01, *I*^2^ = 5.9%) (Table 2). Sensitivity analysis (Figure 3B for the dominant model) was conducted and the results were stable on the whole. Cumulative analysis (Figure 3C for dominant model) demonstrated a significant protective association from the study of Zhang et al. in 2015 [32]. No publication bias was found in the funnel plot and Egger's test (C vs. G, *P* = 0.80; CC vs. GG, *P* = 0.55; GC+CC vs. GG, *P* = 0.61; CC vs. GG+GC, *P* = 0.19) (Figure 3D for the dominant model).

### For the +1612 G/A polymorphism

**Figure 4:**
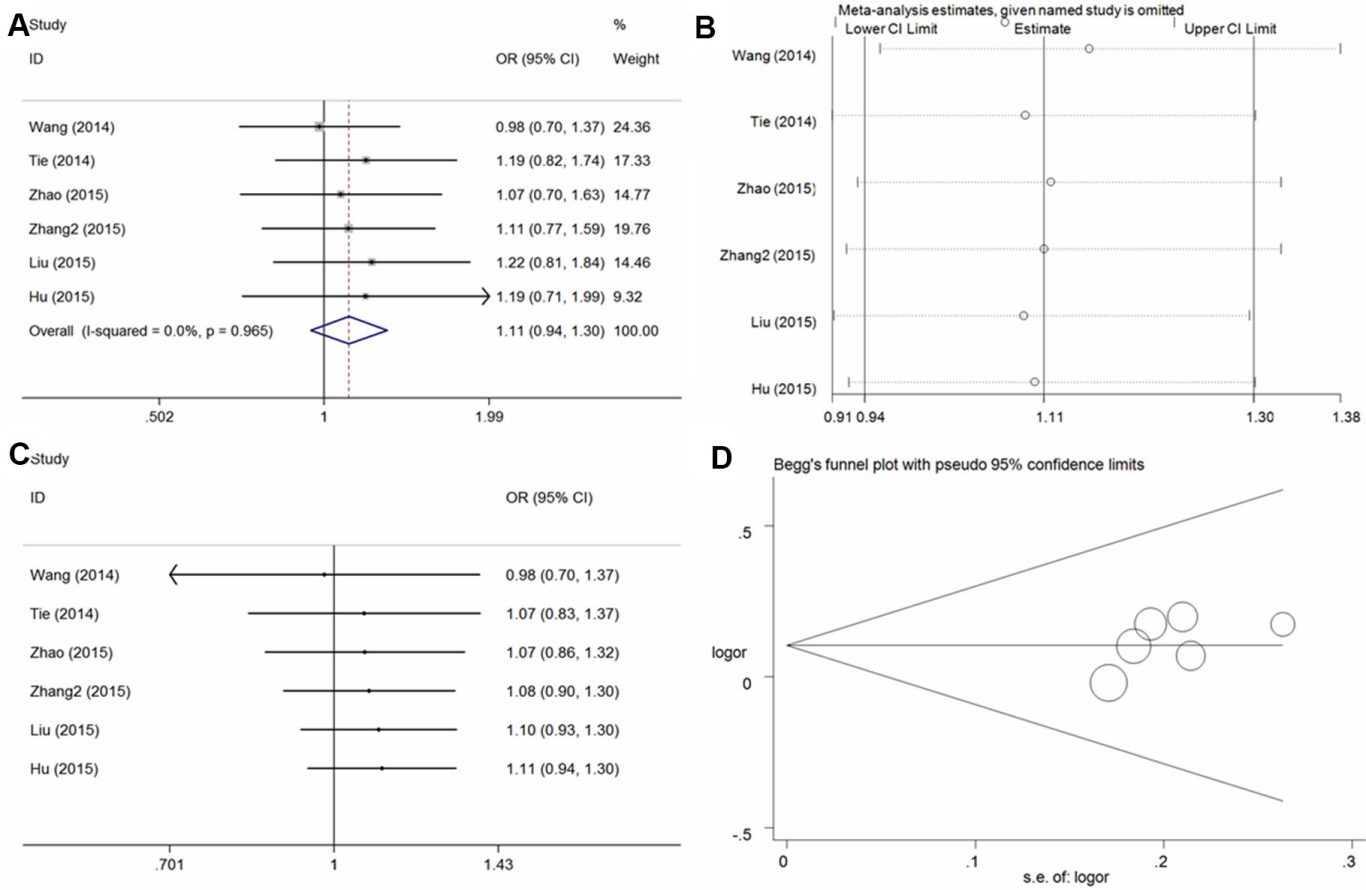
Statistical analysis of the association between the *VEGF* +1612G/A polymorphism and osteosarcoma risk in the GA + AA vs. GG model (**A**) ORs and 95% CIs; (**B**) sensitivity analysis; (**C**) cumulative analysis; (**D**) publication bias.

In the six studies included, no significant association with osteosarcoma risk was found in any model in the total population(A vs. G: OR = 1.10, 95% CI = 0.98–1.23, = 0.10, *I*^2^ = 0%; GA vs. GG: OR = 1.07, 95% CI = 0.91–1.27, = 0.40, *I*^2^ = 0%; AA vs. GG: OR = 1.21, 95% CI = 0.95–1.54, *P* = 0.12, *I*^2^ = 0%; GA + AA vs. GG: OR = 1.11, 95% CI = 0.94–1.30, *P* = 0.21, *I*^2^ = 0% [Figure 4A for the dominant model]; AA vs. GG + GA: OR = 1.17, 95% CI = 0.95–1.46, *P* = 0.15, *I*^2^ = 0%) and subsequent subgroup analysis (Table 2). Sensitivity analysis (Figure 4B for dominant model) and cumulative analysis (Figure 4C for the dominant model) were conducted, and no substantial changes in the ORs were observed. Furthermore, no publication bias was detected, suggesting that the results are statistically robust (A vs. G, *P* = 0.17; GA vs. GG, *P* = 0.25; AA vs. GG, *P* = 0.19; GA + AA vs. GG, *P* = 0.21; AA vs. GG + GA, *P* = 0.54) (Figure 4D for the dominant model).

## DISCUSSION

Angiogenesis is a critical cause of proliferation, invasion, and metastasis in cancer [36]. VEGF is one of the most important cytokines in angiogenesis, which can promote the mitosis of vascular endothelial cells and accelerate the formation of new blood vessels [37]. High expression of VEGF in the primary tumor has been found in certain types of malignant tumors with high metastatic rates and poor prognoses, such as breast, esophageal and colorectal cancers [38, 39].

Studies of osteosarcoma have strongly suggested that higher VEGF expression and activity in primary tumor tissue correlates with increased local microvessel density, faster development of pulmonary metastasis, and poorer prognosis for osteosarcoma patients [40, 41]. An increasing number of studies are focusing on the associations among gene mutations (particularly SNPs), protein expression/activity, and tumor occurrence. Wang et al. [27] conducted the first case-control study of the *VEGF* gene and reported that the +936C/T polymorphism was associated with increased osteosarcoma risk in a Chinese population (T vs. C: OR = 1.31, 95% CI = 1.02–1.68, *P* = 0.04; TT vs. CC: OR = 2.70, 95% CI = 1.34–5.45, *P* < 0.01). Since then, a series of case-control studies have been conducted, but the conclusions have been inconsistent. In 2016, Zhang et al. [42] conducted a meta-analysis and observed that *VEGF* −634 G/C and +936 C/T polymorphisms were significantly associated with osteosarcoma risk. However, their meta-analysis only included three case-control studies. To date, four additional studies have explored the associations between *VEGF* polymorphisms and osteosarcoma. In our meta-analysis of seven eligible case-control studies, more precise associations between the three most common polymorphisms in *VEGF* (+936 C/T, −634 G/C, +1612 G/A) and osteosarcoma susceptibility were investigated than in the previous meta-analysis. All the results revealed that the *VEGF* +936 C/T polymorphism significantly increased the risk of osteosarcoma in the Chinese population. Subgroup analyses based on the HWE status and control design also identified similar risks. Interestingly, our results suggested that the VEGF −634 G/C polymorphism protects against osteosarcoma development. However, no significant association between *VEGF* +1612 G/A and osteosarcoma susceptibility was observed. These inconsistent findings among the three SNP loci indicat that different polymorphisms exert different effects on gene function, even when they are located at the same unit.

Meta-analysis is an effective method of combining the quantitative results of previous studies in order to derive a pooled summary conclusion through statistical measures [43, 44]. This can reduce the risk of drawing incorrect conclusions based on insufficient methods or small sample sizes. We conducted this meta-analysis to comprehensively investigate the relationships of *VEGF* polymorphisms (+936C/T, − 634 G/C, and +1612 G/A) with osteosarcoma susceptibility. None of the studies evaluated herein displayed significant heterogeneity or publication bias within the three common polymorphism loci. All the pooled data yielded consistent results, which not only confirms the validity of our results, but also supports our statistical methods.

However, there were some limitations to our meta-analysis. First, only seven eligible studies were collected, and the limited number of studies with small sample sizes may have affected the analysis of the correlation between *VEGF* polymorphisms and osteosarcoma susceptibility. Second, all the included studies were conducted with Chinese subjects. Ethnicity bias may exist because a single Asian race was examined, and the conclusions may not be applicable to other races. Third, the interactions among various risk factors, such as smoking, drinking, and other genetic factors, are crucial determinants of cancer formation. Thus, the inherent mechanism could not be explained clearly with unadjusted databases in this meta-analysis. Fourth, deviations from HWE were found in the distribution of controls in some of the included studies, possibly due to the insufficient of sample sizes or genotyping errors in those studies.

Our meta-analysis contained a large sample size, new research data and high-quality statistics. The results are credible and reliable as a preliminary exploration of the relationship between the *VEGF* polymorphisms and osteosarcoma susceptibility, and suggest that analysis of *VEGF* polymorphisms may be useful for the early clinical diagnosis and treatment of osteosarcoma.

In summary, our meta-analysis suggests that *VEGF* polymorphisms are associated with osteosarcoma susceptibility in the Chinese population. Nevertheless, additional studies including different ethnicities and larger populations are needed for further exploration of these associations.

## MATERIALS AND METHODS

### Search strategy

Three online electronic databases (PubMed, Embase, and Science Citation Index) were searched with the following terms: (“osteosarcoma”) AND (“vascular endothelial growth factor” OR “VEGF”) AND (“polymorphism” OR “SNP” OR “single nucleotide polymorphism” OR “variant”), from inception to January 2016. Furthermore, all the references of the collected studies were examined so that additional relevant studies could be identified. Only English language full-text case-controls studies were included.

### Study selection

All the selected studies met the following inclusion criteria: (1) case-control studies focused on osteosarcoma; (2) reported on *VEGF* polymorphisms; (3) evaluated the association between *VEGF* polymorphisms and osteosarcoma risk; (4) presented adequate genotype data or data necessary to calculate the OR and 95% CI; and (5) described SNP loci which were reported in at least five publications. Studies were excluded if they: (1) were review papers; (2) were animal studies; (3) contained insufficient information for assessment of the association; (4) contained repeated or overlapping results from another study (in such cases, only the study with the largest sample size or the most recent study was included).

### Data extraction

Two reviewers (Hu and Du) independently conducted data extraction using a standardized form. The following information was collected: the first author's name, year of publication, sources of controls, study region or country, ethnicity of subjects (such as Asian or Caucasian), genotyping method and genotype distribution data for cases and controls. Disagreements were resolved by a third author during the analysis.

### Statistical analysis

HWE was evaluated with the chi-square test using the genotypes of the controls. The association between the genotype distribution and osteosarcoma was evaluated based on the ORs and corresponding 95% CIs. For example, the pooled ORs were obtained for the allele contrast (T vs. C), co-dominant model (CT vs. CC, TT vs. CC), dominant model (CT + TT vs. CC), and recessive model (TT vs. CC + CT) in the *VEGF* +936C/T locus. Similar genetic models were also assessed for the other SNPs. Heterogeneity was assessed with Cochran's Q statistic and the *I*^2^ method, and *P* < 0.10 or I^2^ > 40% were considered to demonstrate substantial heterogeneity [45]. ORs were estimated with a fixed-effects model (the Mantel-Haenszel method) when there was no considerable heterogeneity [46]; otherwise, a random-effects model (DerSimonian and Laird method) was adopted [47]. Subgroup analyses were conducted based on the classification of the study design and HWE status. Cumulative meta-analyses were conducted so that any potential trends in the pooled estimates over the study years could be identified. Sensitivity analyses were conducted, in which the stability of the results was evaluated as each study was sequentially removed for each locus. Egger's linear regression and Begg's funnel plots were used to assess potential publication bias. Statistical analysis was performed with STATA version 12.0 (Stata Corporation, College Station, TX, USA) with two-sided *P* values, and *P* < 0.05 was considered statistically significant.
